# Examination of the Respiratory System

**Published:** 1913-07-05

**Authors:** 


### THE SCHOOL DOCTOR.

### V.—Examination of the Respiratory System.

Some of the most difficult problems that the school doctor has to decide are those concerning the integrity of the school child's lungs. It is not often that a definite diagnosis of pulmonary tuberculosis can be confidently made, for usually these advanced cases, in which there is no doubt whatever about the disease, go to a hospital and do not come under the school doctor's observation. It is the incipient case that he meets with, and it is just with regard to the incipient case that he has to exercise great caution and to be thoroughly well acquainted with the lung signs in the normal child so as to be able to note what is abnormal.

In examining the child's chest, the school doctor should proceed with the same systematic regularity and detail that he employs in his hospital cases. Usually it is easy enough to be certain in one's mind that the lungs are normal. In such a case the examination takes only a few minutes, and unless there is reason to suspect that a further examination is necessary the examiner may proceed to another system. But sometimes this methodical examination of the thorax reveals a finding that strikes the examiner as somewhat unusual, and then it is often a matter of some difficulty to class the child properly. The most common difficulty is undoubtedly with regard to tuberculosis of the lungs. It is a well-known fact, with which every student who has attended demonstrations in the post-mortem room is familiar, that the adult lung is very rarely found to be absolutely free from all traces of old tuberculous mischief. So common, indeed, is it to find such traces that Rosin has laid down the axiom that "Tuberculosis of the lungs is in general, and almost as a rule, to be reckoned as a harmless affection which can scarcely be called a disease." While one may not be prepared to accept this dictum in its entirety, few of us will oppose the premiss that there is a marked difference between what may be called "anatomical tuberculosis" and "clinical tuberculosis" of the lungs—that is to say, that detailed examination of the chest may often elicit physical signs which have not given, and probably will not give, rise to symptoms sufficiently serious to warrant the diagnosis of phthisis. It is just as well, as a preliminary, to discuss what these signs are.

These signs are found, almost invariably, in the right apex, for the deviations from the normal in the left apex are extremely rare. It is generally taught to students that the normal breathing over the right apex agrees with that heard over the left, that is to say, the breathing is purely vesicular, with a short and ill-defined expiration note, bronchial breathing being only heard, and not invariably, when the region adjoining the trachea is auscultated. As a matter of fact no such rule can be laid down with regard to the right apex. Out of every hundred cases of presumably normal children, sixty show appreciable variations in regard to the breathing, and even in regard to the percussion findings, over the right apex. With light percussion it is often possible to demonstrate that there is a slight difference between the two apices, the note over the right being less resonant than that over the left. Three different types of respiration are almost normal over the right apex: one in which the expiration note is prolonged, without change in the inspiration; the second in which, while the inspiration note is vesicular and unchanged, the expiration is prolonged and at the same time roughened, approaching the bronchial type; and, thirdly, where the expiration note is bronchial in type while the inspiration note is shortened and ill-defined. These three different types of breathing may be found in children who are clinically perfectly healthy; they are also found in cases in which there is undoubtedly commencing tubercular mischief at the apex. It is, therefore, of the highest importance to amplify the auscultatory findings by other evidence before a correct diagnosis can be arrived at.

Minor variations in the percussion note, accompanied with one or other of these three types of breathing, cannot by itself be taken as a proof that the right apex is diseased. Nor is it likely that the school doctor has at hand the refinements of diagnostic technique which the hospital physician can employ, while it is doubtful if such refinements—the use of the $x$-rays for example—can clinch the diagnosis. The best test is, after all, the general clinical state of the child. A child presenting such abnormal apical variations should have its temperature and weight methodically and carefully registered. Some care must be exercised in taking the temperature by the mouth, and allowance must be made for the well-known variations which occur in children owing to psychical and emotional causes. For these reasons it is always preferable to have the average of three different measurements taken successively. It is quite useless to depend on a half-minute thermometer for these measurements; five-minute measurements must be taken (some authorities suggest a minimum of ten minutes, which is certainly preferable), and it is well to take temperatures while the child is resting and compare them with temperatures measured when the child has had some exercise, such as running, jumping, etc. Temperatures must be taken in the morning, at mid-day, and in the late afternoon; when systematically registered they give the best indication of the fitness of the child. For example, a rise in temperature, provided it is regular and not subject to fluctuations ascribable to emotional causes, is fair evidence on which to base the assumption that the physical signs detected in the right apex are pathological. Progressive loss of weight, anæmia, and malnutrition, are additional danger signs, and when they are accompanied by such symptoms as night sweats, lassitude, and headache, there is usually not much doubt as to the seriousness of the condition present. Finally the tuberculin reactions may be tried. These cannot be administered at the school, but with the findings at examination, supported by the additional data obtained by careful watching and registration of temperature, the school doctor is justified in recommending that such a child should attend a tuberculosis dispensary for confirmatory tests.

Where these variations in the breathing are heard over the right apex, during the routine examination of school children, it is, therefore, well for the examiner to make out a "doctor's" card to enable him to follow up these cases for his own satisfaction. In the majority of cases such children go through their school career without developing signs of phthisis, but we have not sufficient data to say what their future life is likely to be. It would be an interesting study to trace the after-school history of such cases, in order to find out whether these variations ultimately develop into active signs of tuberculosis. Judging from post-mortem findings one can only state that it appears that they do not mean that the child is actively tuberculous, and that their significance is therefore far less ominous than is usually stated to be the case.

